# Elasticity spectra as a tool to investigate actin cortex mechanics

**DOI:** 10.1186/s12951-020-00706-2

**Published:** 2020-10-20

**Authors:** Ines Lüchtefeld, Alice Bartolozzi, Julián Mejía Morales, Oana Dobre, Michele Basso, Tomaso Zambelli, Massimo Vassalli

**Affiliations:** 1 Laboratory of Biosensors and Bioelectronics, ETH Zürich, Gloriastrasse 35, 8092 Zürich, Switzerland; 2 Dipartimento di Ingegneria dell’Informazione, Università degli studi di Firenze, Via di S. Marta 3, 50139 Firenze, Italy; 3 Institut de Physique de Nice, Université Côte d’Azur, 1361 Route des Lucioles, 06560 Valbonne, France; 4 Dipartimento di Medicina Sperimentale, Università degli studi di Genova, Via Leon Battista Alberti 2, 16132 Genova, Italy; 5 James Watt School of Engineering, University of Glasgow, Oakfield avenue, Glasgow, G12 8LT UK

**Keywords:** Scanning probe microscopy, Force spectroscopy, Cell mechanics, Nanoindentation, Cytoskeleton, Actin cortex

## Abstract

**Background:**

The mechanical properties of single living cells have proven to be a powerful marker of the cell physiological state. The use of nanoindentation-based single cell force spectroscopy provided a wealth of information on the elasticity of cells, which is still largely to be exploited. The simplest model to describe cell mechanics is to treat them as a homogeneous elastic material and describe it in terms of the Young’s modulus. Beside its simplicity, this approach proved to be extremely informative, allowing to assess the potential of this physical indicator towards high throughput phenotyping in diagnostic and prognostic applications.

**Results:**

Here we propose an extension of this analysis to explicitly account for the properties of the actin cortex. We present a method, the Elasticity Spectra, to calculate the apparent stiffness of the cell as a function of the indentation depth and we suggest a simple phenomenological approach to measure the thickness and stiffness of the actin cortex, in addition to the standard Young’s modulus.

**Conclusions:**

The Elasticity Spectra approach is tested and validated on a set of cells treated with cytoskeleton-affecting drugs, showing the potential to extend the current representation of cell mechanics, without introducing a detailed and complex description of the intracellular structure.
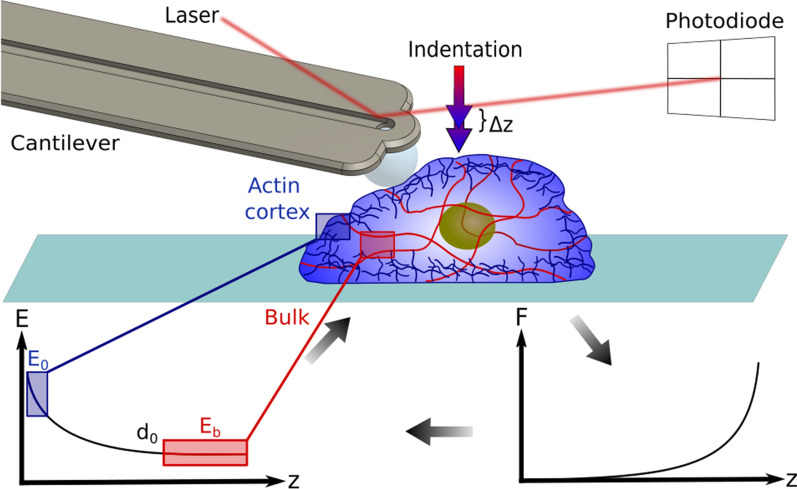

## Background

Every living organism is made of cells that constantly adapt their phenotype to the environment, tuning their biochemical and physical properties to respond to external cues. A central role in this mechanism has been clearly recognized for the mechanical properties, either of the cell or the extracellular environment. The elasticity of the substrate can drive the differentiation of stem cells towards a specific lineage [1], through the engagement of a “molecular clutch” mechanism [2] which is also thought to transduce local viscosity information [3]. On a different perspective, the mechanical properties of cells reflect their physiological state, and measuring the deformability of single cells with high throughput holds a great promise for future diagnostic and therapeutic applications [4, 5]. Altogether, the mechanical interplay between living cells and their environment is a key process, potently involved in the development of organ and organism, and the dysregulation of its homeostasis contributes to the onset of pathological states [6]. As a matter of fact, the number of genetic mutations recorded in cancers has been found to be proportional to the elasticity of the tissue of origin [7], and the invasive potential of single cancer cells is associated to their ability to adapt to the mechanical properties of the surrounding matrix [8].

To better understand and characterize the process of cellular mechanotransduction, it is mandatory to focus the investigation on the cortical region of cells [9], the actin cortex (AC) forming the boundary between the cell body and the extracellular matrix (ECM). The AC consists of the plasma membrane and the underlying actin cytoskeleton, linked together by a rich pool of transmembrane and adaptor proteins [10]. The structure and biomechanics of the AC are tightly intertwined [11, 12] and they in turn influence the functionality of molecular mechanosensors, such as Piezo mechanosensitive ion channels [13, 14] or G-protein coupled receptors [15, 16]. These are incorporated into the plasma membrane and directly convert mechanical stimuli into downstream biochemical signals. There is a growing interest for the identification of assays and methods able to assess the mechanotransduction state of single cells, and exploit it with higher throughput, towards the identification and screening of new drugs [17]. In this view, a simple and reliable method to characterize the physical state of the AC would be a valuable tool to identify innovative label-free biophysical markers of the cellular phenotype [18–20].

The study of AC structure and mechanics is particularly challenging from the technical point of view. The actomyosin network belonging to the AC spans a thickness of few 100nm, outside of the resolution achievable with standard fluorescence microscopy. Few methods have been proposed to quantify it, either using super-resolution microscopy [21, 22] or a smart localization-based technique [23]. On a different note, the mechanics of the AC has been more largely addressed [24], trying to elucidate its peculiar rheological properties [25], and to decouple the contribution of the network elasticity from the cortical tension [26] or active myosin-driven stresses [11, 27]. The richness and complexity of this thin and heterogeneous layer have been largely challenged using micropipette aspiration, a method that provides very controlled measurements, but requires a custom setup and specialized technical skills [28]. A more suitable and scalable approach is based on nanoindentation experiments, exploiting atomic force microscopy (AFM) [29] or recently introduced cantilever-based devices, such as ferrule-top [30], that offer improved usability [31]. The potential of nanoindentation to measure the overall mechanical properties of single cells is nowadays established [32], and the technology is rapidly growing to provide higher experimental throughput [33]. Nevertheless, only few extensions of the analysis have been proposed to explicitly account for the contribution of the AC to cell mechanics [24, 34] and none of them emerged as a consolidated and broadly adopted approach.

Here we present a method to characterize the AC of living cells based on nanoindentation measurements. The proposed analysis is intended to assess the mechanical properties of the cortex to its simplest approximation, describing the cell as an elastic bilayer and using an extension of the Hertzian contact mechanics to obtain the thickness and stiffness of the AC. The proposed methodology is tuned against numerical data and tested on a soft hydrogel. The effectiveness of the overall procedure is validated on single cells treated with drugs affecting the cytoskeleton organization.

## Results and discussion

**Fig. 1:**
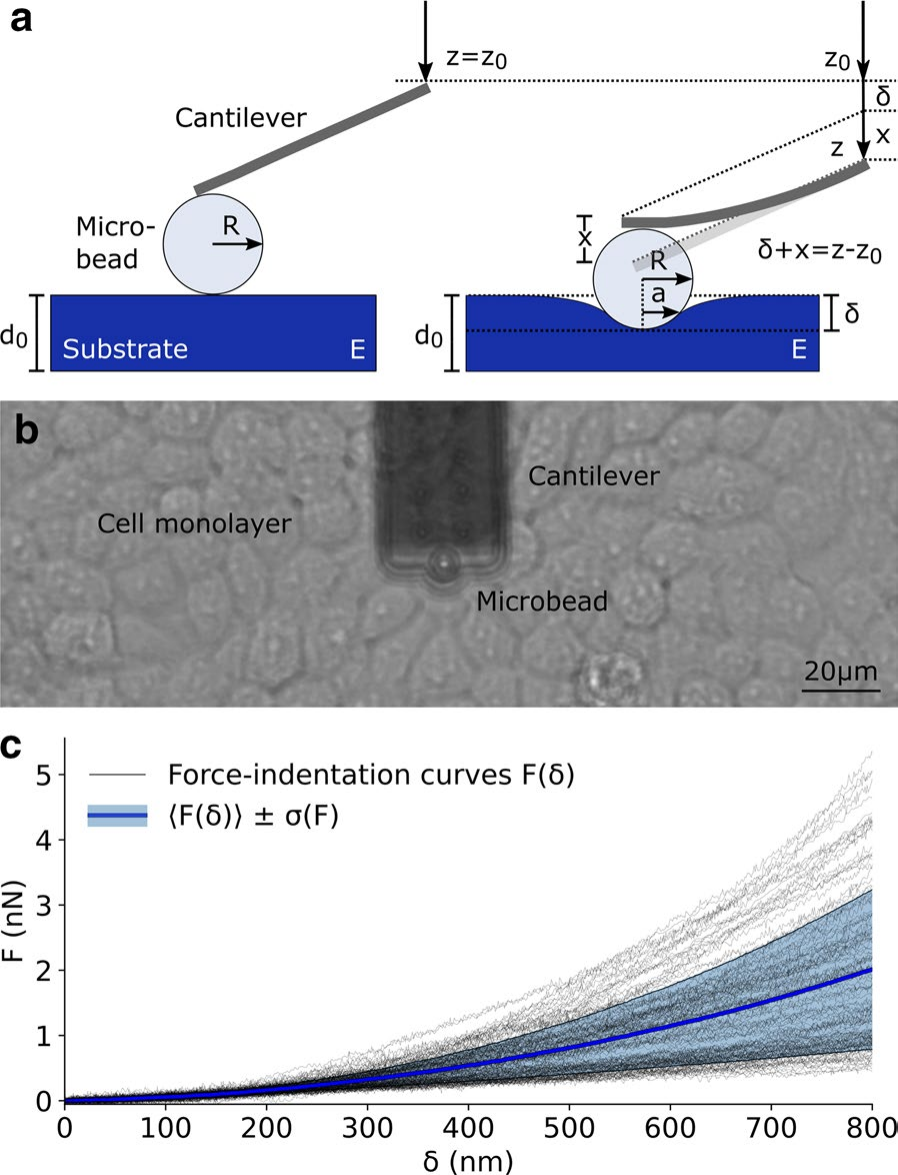
Overview of a nanoindentation experiment. **a** Schematics of a nanoindentation experiment performed with a spherical probe of radius *R* indenting over a compliant material with Young’s modulus *E*. The main geometrical relationships between the displacement *z*, indentation $\delta$ and deflection *x* are indicated. **b** FluidFM cantilever with attached microbead over a confluent monolayer of HEK-293T cells. **c** Typical experimental dataset of 127 force-indentation curves obtained on cells. The blue line is the average curve $\left\langle F(\delta ) \right\rangle$, and the cyan band is extended over one standard deviation

Single cells are complex composite materials [35], integrating static (solid-like) and time-dependent (fluid-like) mechanical components that contribute to the short-term response and long-term adaptability of cells, respectively. Here we concentrate the analysis on the measurement of the elastic component, associated to the tensional state of the cortex that directly influences the functionality of mechanosensitive ion channels [14] and the rapid transport of mechanical forces across the cell [36, 37]. Cell mechanics has been largely studied with cantilever-based nanoindentation, and the limits of validity of the approach have been broadly discussed [38]. The viscous component is expected to impact on the measurements but it has been demonstrated that, if the experimental parameters are carefully controlled, cell mechanics can be robustly described in terms of a single stiffness parameter [29, 31, 39]. The simplest and most adopted approach consists of treating the cell as a homogeneous and isotropic material described by the Young’s modulus *E* which is calculated from single cell indentation curves. With reference to Fig. 1, the experimental force-distance curve *F*(*z*) is converted in a force-indentation curve $F(\delta )$ after the identification of the contact point $z=z_0$. Given that the curve *F*(*z*) is flat and smooth across this point, the identification of $z_0$ is particularly challenging [40]. Many approaches have been proposed to face this issue and broadly benchmarked in the literature [41, 42]. Here we selected a method based on the ratio of variances, that accounts for the change in noise content of the signal before and after contact, without any specific assumption on the expected polynomial trend of the $F(\delta )$ curve [43] (see “Methods” section). A typical experimental result is depicted in Fig.1c where a set of 127 single cell $F(\delta )$ curves is represented, including the average curve (blue) and the standard deviation (shaded region).

### Calculating elasticity spectra from nanoindentation experiments

**Fig. 2:**
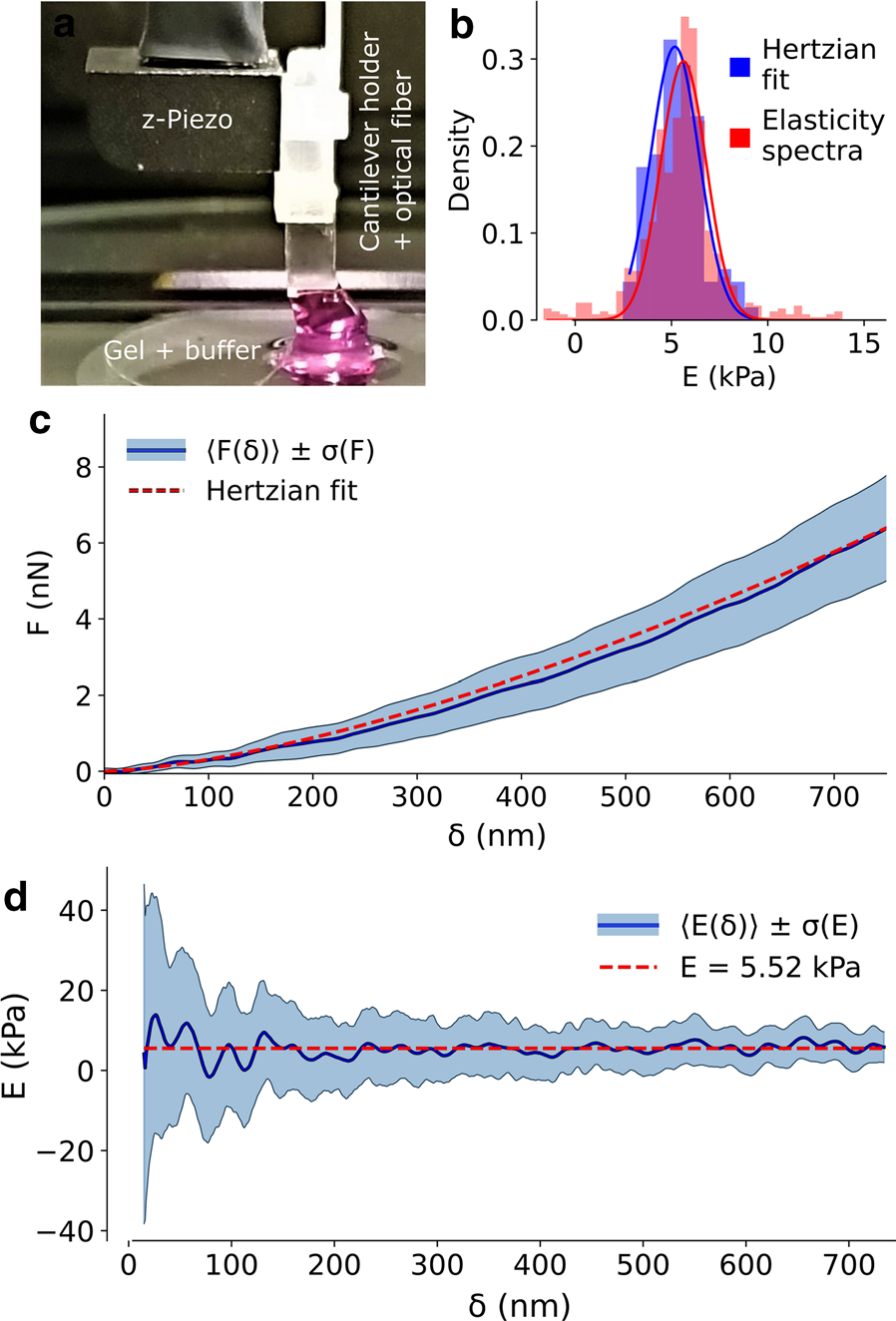
Results of ferrule-top nanoindentation experiments on a homogeneous gel. **a** Picture of the experimental setup, showing the Chiaro nanoindenter with the cantilever holder and optical fiber (white) attached to the z-Piezo (black). The probe with a microbead (transparent) is positioned above the gel and immersed in buffer solution (pink). **b** Histograms of the Young’s modulus as calculated either with the standard Hertzian fit approach (blue) or based on the average elasticity spectrum (red). Gaussian fit of the histogram values provides (peak position ± SEM) $E=5.2\pm 0.2$ kPa for the Hertz approach and $E=5.6\pm 0.1$ kPa for ES. **c** Force-indentation results, showing the average force-indentation curve $\left\langle F(\delta ) \right\rangle$ (blue) and Hertzian fit (red dashed) from a set of 97 nanoindentation experiments. (d) Elasticity spectra analysis corresponding to the curves in **c**, showing the average elasticity spectrum $\left\langle E(\delta ) \right\rangle$ (blue) and its mean value (red dashed)

The current–simplified but effective–approach for obtaining the Young’s modulus from single nanoindentation curves is based on linear contact mechanics [44]. For a spherical indenter, this theory is reduced to the well-known Hertz equation [45]:

1 $$\begin{aligned} F=\frac{4}{3}\frac{E}{1-\nu ^2}\sqrt{R}\delta ^{\frac{3}{2}} \end{aligned}$$

where *F* is the force, $\delta$ the indentation, *R* the radius of the sphere, *E* the Young’s modulus and $\nu$ the Poisson’s ratio of the indented material. Using this equation, it is possible to fit the $F(\delta )$ curves and obtain *E* as a fitting parameter. Figure 2 shows the results of this approach for ferrule-top nanoindentation [30] on a simple homogeneous gel (see “Methods” section); the average of experimental curves is presented in panel (c) while the corresponding elasticity values of single curves is depicted in the blue histogram in panel (b), showing a Gaussian shape centered at $E=5.2 \pm 0.2$ kPa. A similar analysis can be performed following the generalization of the contact mechanics theory proposed by Oliver and Pharr to account for any indentation between a rigid, axisymmetric punch and an elastic half space [46]. For a spherical indenter, the Oliver-Pharr equation has the form:

2 $$\begin{aligned} \frac{dF}{d\delta }=\frac{2}{\sqrt{\pi }}\sqrt{A}\frac{E}{1-\nu ^2}=2a\frac{E}{1-\nu ^2} \end{aligned}$$

where *A* is the contact area, approximated as a circle of radius *a* (see Fig. 1a). The Poisson’s ratio $\nu$ of a cell is a complex frequency-dependent quantity [25]. Nevertheless, if the experimental protocol is not changed between measurements, $\nu$ remains constant and impacts only as a coefficient. For the sake of simplicity, we can adopt the common hypothesis that cells behave as incompressible elastic solids, for which $\nu =0.5$. Under this assumption, Eq. (2) can be further simplified, and the Young’s modulus can be expressed as:

3 $$\begin{aligned} E=\frac{1-\nu ^2}{2a}\frac{dF}{d\delta }=\frac{3}{8a}\frac{dF}{d\delta } \end{aligned}$$

The original solution of Sneddon suggests that for a spherical indenter of radius *R* the contact radius *a* is simply calculated as [44]:

4 $$\begin{aligned} a=\sqrt{R\delta } \end{aligned}$$

The validity of Eq. (4) has recently been validated for living cells, in a typical range of experimental parameters [29]. By substituting this expression in Eq. (3) we obtain the final expression:

5 $$\begin{aligned} E=\frac{3}{8\sqrt{R}}\frac{1}{\delta }\frac{dF}{d\delta } \end{aligned}$$

This equation is expected to provide the same results as Eq. (1), even though experimental noise and numerical aspects can lead to non-negligible discrepancies [47]. The set of force-indentation curves acquired on a homogeneous gel in Fig. 2c was analyzed with this alternative approach. Using Eq. (5) on each individual curve, a set of curves for *E* as a function of the indentation $\delta$ is obtained, namely the elasticity spectra (ES). The average ES, $\left\langle E(\delta )\right\rangle$, is depicted in blue in Fig. 2d. To compare this alternative approach to the standard Hertzian one, based on Eq. (1), the values of $\left\langle E(\delta )\right\rangle$ are plotted as a histogram in red in Fig. 2b, showing that the two approaches provide highly comparable results.

### Bilayer model

**Fig. 3:**
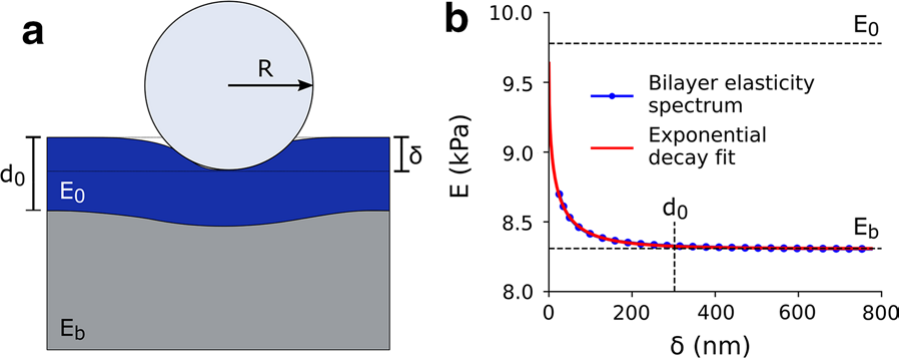
Bilayer model. **a** Schematics of the geometry of the bilayer model, with an external layer with elasticity $E_0$ and thickness $d_0$ and a bulk substrate with elasticity $E_b$, indented with a sphere of radius *R*. **b** Elasticity spectrum (blue dots) calculated from numerical data for a bilayer with $E_0$ = 9.8kPa, $d_0$ = 300nm and $E_b$ = 8.4kPa indicated by the black dotted lines. The solid red line represents the exponential fit that returns the values $E_0$ = 9.77kPa, $d_0$ = 302nm and $E_b$ = 8.39kPa

The simplest model, by which a cell is described as a homogeneous material of Young’s modulus *E*, has been effectively exploited in applications, even though it neglects many aspects of the inner structure of the cell. Several models have been suggested to account for the heterogeneity of the system, often resulting in a detailed but complex description of the cell mechanics that did not translate to any broader use. Here we want to extend the successful Hertzian approach in order to account for the AC in the simplest geometry. To this purpose, we describe the cell as a bilayer (see Fig. 3a) with an external layer of thickness $d_0$ and elasticity $E_0$ (representing the AC) sitting on top of an indefinitely thick softer substrate of elasticity $E_b<E_0$. When the force-indentation curve of such a system is described by standard contact mechanics, it is expected to exhibit a depth-dependence of the Young’s modulus. This effect is experimentally well known [48, 49], and Finite Element Analysis (FEA) approaches suggest that this behavior is potently affected by the presence of a stiffer cortex, more than by experimental artefacts or other components, which is highlighted when using sharp indentors [50]. To isolate the contribution of the AC to the experimental force spectroscopy curves, it is crucial to characterize this depth-dependent phenomenon. The standard approach to analyze nanoindentation measurements is based on a fit of the experimental data to Eq. (1). This method exploits the robustness of the fit against noise to provide a solid reference value for the Young’s modulus over the fitting range, but at the same point any dependence of *E* from the indentation $\delta$ is averaged out. Changing the maximum indentation $\delta _{max}$ used for the fitting range, a curve $E(\delta _{max})$ is obtained that can be interpreted as an apparent modulus [51]. Nevertheless, fitting over a range provides a sort of convolution between different layers, and the sensitivity of the method to local variations is low by design. Instead, we propose to use the approach based on Eq. (5), to obtain elasticity spectra from the local slope of the force-indentation curve (see “Methods” section for details). This method is intrinsically more sensitive to the noise, being differential instead of integral, but in turn it provides a greater sensitivity to depth variations.

The bilayer problem has been studied in the literature, and a general analytic expression for $E(\delta )$ is not available. Nevertheless, a nanoindentation experiment on a bilayer has been simulated using FEA, obtaining a numerical expression for $F(\delta )$ that matches the experimental results and can be well approximated with a polynomial equation where the coefficients are calculated for a specific range of the physical parameters [52]. Doss et al. tested this solution on a set of layered gels with controlled elasticity, in a region of the parameters that cannot be directly translated to the case of the AC, but it demonstrates the validity of the numerical approach. Here we extended the same simulation approach to obtain a set of numerical $F(\delta )$ curves and exploited Eq. (5) to calculate the corresponding ES. The blue dots of Fig. 3b show the results obtained for an arbitrary set of the parameters $E_0$, $E_b$ and $d_0$ compatible with cellular values ($E_b<E_0$ ; $d_0<< R$). Figure 3b highlights the expected decay of the elasticity from the cortex to the bulk, over an indentation depth comparable with $d_0$.

While no analytical solution exists for the bilayer problem, some empirical expressions have been identified in the literature, where the decay has been described by a generalized form [53]:

6 $$\begin{aligned} E(\delta )=E_b+(E_0-E_b)\phi (\delta ) \end{aligned}$$

where the function $\phi (\delta )$ decays from 1 to 0 over a range related to $d_0$. In particular, it has been shown that either a trigonometric [54] or exponential [55, 56] decay well approximates the experimental behavior. These two phenomenological descriptions are very similar, and the exponential one provides a cleaner and simpler analytical equation:

7 $$\begin{aligned} \phi (\delta )=e^{-\frac{\Lambda a}{d_0}}=e^{-\frac{\Lambda \sqrt{R\delta }}{d_0}} \end{aligned}$$

where *R* is the radius of the spherical indenter and $\Lambda$ is a phenomenological parameter. Eq. (6) with the weight introduced in Eq. (7) was used to fit the numerical data of Fig. 3 (solid red curve).

**Fig. 4:**
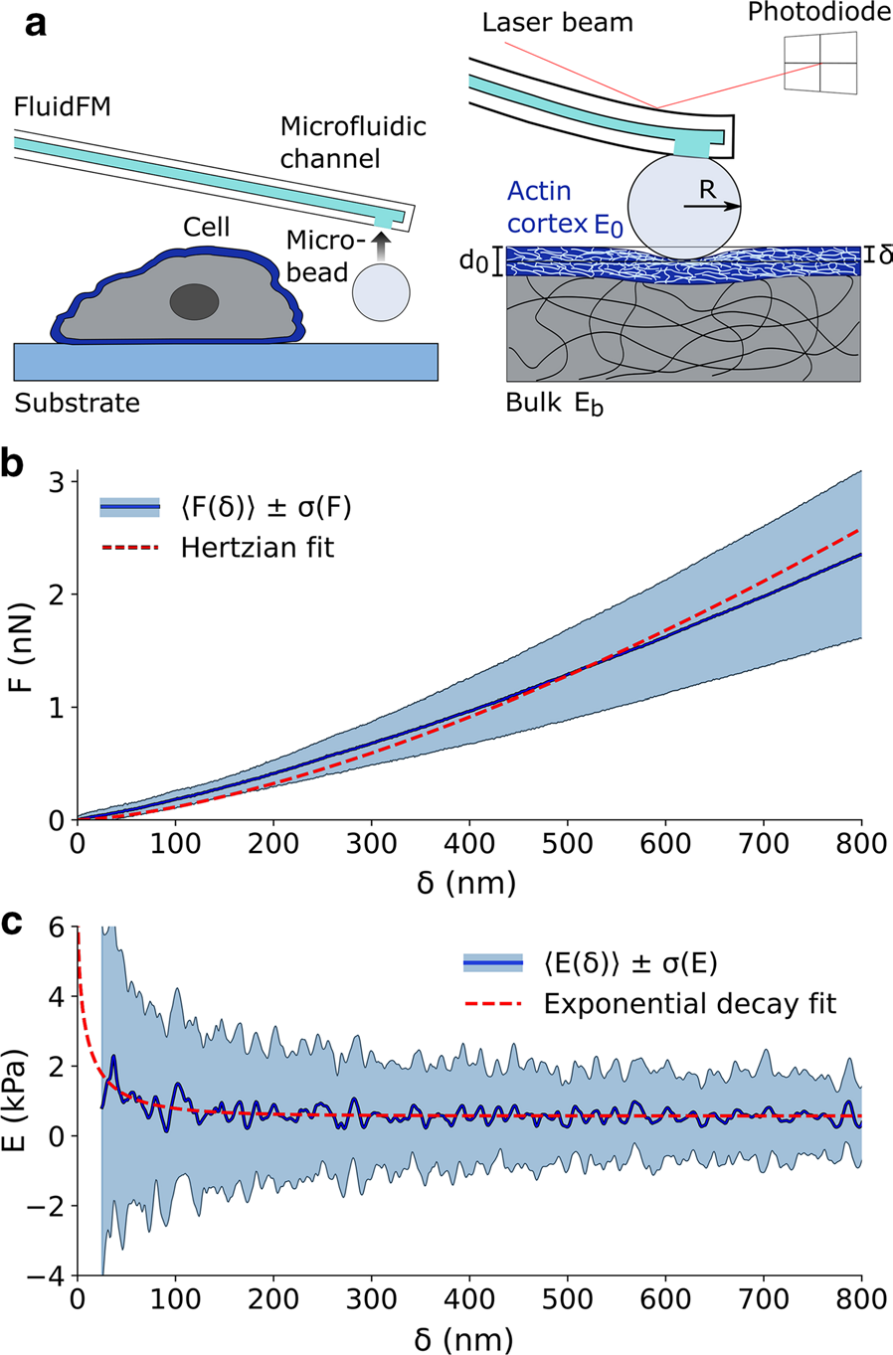
Elasticity spectra of single cells. **a** Schematic of the experimental protocol: a microbead is collected with the FluidFM cantilever and used to indent the cell, described as a double layer with the external AC of thickness $d_0$ and elasticity $E_0$ and the cytosol with bulk elasticity $E_b$. **b** Experimental set of force–indentation curves $F(\delta )$ obtained on 315 cells. The blue line indicates the average curve and the red dashed line is the best Hertz fit (Eq. 1). **c** Elasticity spectra obtained by the application of Eq. (5) to the single curves of **b**. The blue line indicates the average of tghe Elasticity Spectra and the red dashed line is the fit with the exponential bilayer model (Eq. 8)

This approach to describe the indentation of an elastic bilayer can be adopted to offer an effective and simple representation of cell mechanics including the role of the AC. A reference set of single cell nanoindentation experiments obtained by fluidic force microscopy (FluidFM) is presented in Fig. 4 [57]. The inadequacy of the standard Hertz model to describe the average force-indentation curve is apparent in panel (b), where the red dashed line represents the fit based on Eq. (1). Instead, we propose to calculate the elasticity spectra for each curve (cyan band in Fig. 4c) and fit the average spectrum with the exponential bilayer model:

8 $$\begin{aligned} E(\delta )=E_b+(E_0-E_b)e^{-\frac{\Lambda \sqrt{R\delta }}{d_0}} \end{aligned}$$

This equation can be fitted to the data to obtain an estimate of the elasticity of the cortex and the bulk. The red dashed line in Fig. 4c represents the fit obtained with this procedure. While the parameters $E_0$ and $E_b$ are directly obtained from Eq. (8), to retrieve an estimate of $d_0$, the a priori knowledge of $\Lambda$ is required. In order to determine this phenomenological parameter, we used the FEA approach implemented in [52], obtaining a calibration value for this parameter ${\bar{\Lambda }}=1.74$ (see “Methods” section). While a further experimental evaluation of this parameter is envisaged to obtain a nanometer-reliable value of the thickness, it is important to notice that it impacts the results in terms of a scale factor. In other words, while the absolute value of $d_0$ is affected by ${\bar{\Lambda }}$, any relative change is not. Moreover, a numerical investigation of the dependency of ${\bar{\Lambda }}$ from the model parameters suggests that in a physiologically relevant range it is not expected to change by more than about 10% (see Additional file 1: Fig. S2).

### Characterizing the actin cortex with the bilayer decay model

To validate the proposed approach, we applied the analysis to a set of nanoindentation experiments performed on single cells in control conditions and treated with drugs differentially acting on the cytoskeleton. Control HEK-293T cells were characterized through the elasticity spectra approach; Fig. 4 presents a typical experimental output (Fig. 4b), the corresponding ES (Fig. 4c) and the schematics of the experimental procedure (Fig. 4a). From each experimental session, a set of fitting parameters $E_0$, $E_b$, $d_0$ can be obtained through Eq. (5).

**Fig. 5:**
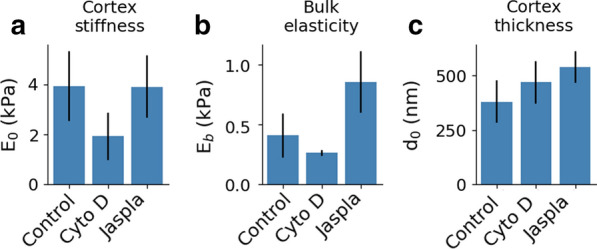
Results of the elasticity spectra analysis for control and drug treated HEK-293T cells. The elasticity of the cortex (**a**), of the cytosol (**b**), and the thickness of the cortex (**c**) were calculated for a set of control cells (N = 4 with 13–36 cells each) and after treatment with either 10 μM cytochalasin D (Cyto D, inhibiting actin polymerization, N = 5; 17–33 cells) or 1 μM jasplakinolide (Jaspla, inducing actin polymerization, N = 4; 20–36 cells). Error bars represent the variance of the repeats, calculated over the weighted average

The bulk modulus $E_b$ corresponds to the asymptotic value of the elasticity for indentations larger than the thickness. Using a maximum depth of around 800nm (see Additional file 1: section Addtional file.1 for further discussion), this value can be evaluated with a very high numerical accuracy. This quantity can be compared with the standard Young’s modulus, obtained using Eq. (1). HEK-293T cells have been widely characterized in literature, and a broad range of values has been declared, from 300 to 400 Pa [58, 59] up to few kPa [60, 61]. This issue in comparing experiments obtained in different conditions has been recently challenged [62], and it is suggested that changes in the mechanical properties—as measured in the exact same conditions—are more relevant than absolute values [31]. Nevertheless, the values obtained using the ES approach (350–550 Pa, see Fig. 5b) lie within the range of published values. In addition to the bulk elasticity, the ES bilayer decay method allows to calculate the parameters of the AC. Some measurements of the thickness of the AC are reported, indicating a value of about 100–200 nm for mitotic cells [10, 23] that increases to 300–400 nm in the adherent phase [63]. The measured values of $d_0$ where in the range 290–470 nm (see Fig.5c), in line with previous results (even though obtained on different cells, and so not directly comparable). The parameter $E_0$ represents the AC stiffness, a parameter that provides information on the elastic properties and is related to the cortical tension [24]. Only few authors provided an estimate of the AC stiffness, which is expected to be up to 10 times harder than the bulk [64], as observed in our experiments on control HEK-293T cells (Fig.5a).

To evaluate the effectiveness of the approach, the same HEK-293T cells were treated with either cytochalasin D or jasplakinolide (Fig. 5). Cytochalasin D is a fungal metabolite [65] known to soften cells in a dose-dependent manner [66] by disrupting structured actin microfilaments and inducing a larger number of free ends [67], but the specific effect on the AC is not known. The overall softening is confirmed by the ES analysis (Fig. 5b), that provides additional insights on the cortex, showing that AC stiffness is even more pronouncedly reduced, by about 50%, and the thickness is slightly increased, by 20% (Fig. 5a, c). Jasplakinolide, a cyclo-depsipeptide that polymerizes and stabilizes actin filaments [68], has been widely used to challenge the physical properties of the AC. Jasplakinolide treatment is known to induce a thickening of the AC that can be as large as 50% [23] and this behavior was confirmed by the ES analysis of HEK-293T cells treated with jasplakinolide, where $d_0$ grows by about 43% (Fig. 5c). The effect of this drug on the cortical tension *T* of mitotic cells has been previously studied, showing a marked reduction of *T* [11]. The approach offered by ES allows to characterize adherent cells, whose AC stiffness does not appear influenced by jasplakinolide (see Fig. 5a), and the integration of the method with micropipette aspiration is expected to offer new insights into the link between cortical tension *T* and (apparent) stiffness $E_0$ [69]. Moreover, cortex elasticity, thickness and bulk elasticity all together contribute to the value of Young’s modulus measured with standard bulk methods (based on Eq. 1). The effect of a drug on this overall parameter is expected to sensibly depend on the experimental conditions (for example the maximum indentation), and this is especially true for jasplakinolide that influences all components in a differential way. This crosstalk could be at the origin of some inconsistency in the existing literature. In fact, while an overall stiffening of the cell has been reported [70], consistent with the stabilization effect of the drug, other authors observed the opposite behavior in the past [71]. One of the main advantages of the proposed ES approach is that these intertwined components of cell mechanics can be evaluated separately.

## Conclusions

In this paper we presented a method for treating nanoindentation curves that relies on the Oliver-Pharr contact mechanics theory (Eq. 2). Instead of analyzing the force-indentation curve, we suggest concentrating on the apparent Young’s modulus as a function of the depth, the elasticity spectrum $E(\delta )$. This view has several technical advantages. First, it provides a direct visualization of the depth dependency of the nanoindentation experiment and it allows for an accurate working range selection. As a matter of fact, the standard contact mechanics analysis is based on the main assumptions that the indentation is small enough to avoid the effect of any underlying substrate and-at the same time-to remain in the range of validity of Eq. (4). This is normally translated to an empirical law that suggests to limit the indentation to about 10% of the smallest between the radius of the indenter and the thickness of the material [72, 73]. Using ES, this limit can be experimentally identified, looking at the behavior, and avoiding large indentations for which the curve starts rising (see Additional file 1: Fig. S1).

Furthermore, the ES can be interpreted based on a constitutive model of the material that accounts for vertical inhomogeneities. In this paper, we presented the treatment for the case of an elastic bilayer, that can be phenomenologically described by an exponential decay in the ES (Eq. 8). The robustness of the approach has been evaluated using two different nanoindentation devices, namely the ferrule-top Chiaro system and an AFM equipped with FluidFM add-on (see “Methods” section). These systems are suitable to optimize the throughput of the experiment, needed to get rid of the higher noise sensitivity of Eq. (5). In particular, the microfluidics of FluidFM can be exploited to pick-up the sphere, and release it after few indentations (in case of contamination), without the need of gluing the sphere on the cantilever in advance, or changing the cantilever for every experiment [57, 74]. On a different note, the interferometric read-out of the Chiaro nanoindenter results in a very quick and practical calibration and set-up of the experiment, without any special requirement for the sample holder (see for example the arrangement pictured in Fig. 2a).

The validated ES method was further applied to study the physical properties of the AC. This compartment has a crucial role in cellular sensing and force transduction, and the measurement of AC-related mechanical indicators has clearly demonstrated a great phenotyping potential in a genome-scale study of single mitotic cells [75]. The ES, together with the exponential decay of Eq. (8), offer a simplified view of the AC, in terms of a bilayer geometry. This model does not consider the finer details of the AC structure and dynamics, nor does it take into account the inherent heterogeneity of the cytosol associated to the presence of intracellular organelles, such as the nucleus, that are knoewn to impact the measured mechanical properties [76]. Nevertheless, it is able to capture the main behavior, offering a simple and effective method to extract mechanical parameters associated to AC and bulk cell mechanics.

The ES approach shares some technical limitations with the standard contact mechanics analysis of cell mechanics. In particular, measuring absolute values, to be compared between completely different experimental settings, is a challenging task [62]. Nevertheless, relative changes are expected to provide a robust and reliable indicator, and the ES-based bilayer model offers a valuable tool for the screening of drugs affecting AC mechanics [11, 17], the evaluation of the effect of environmental or physio-pathological conditions on the dynamics of AC [77–79], and the investigation of the impact of intracellular forces to cell mechanosensitivity [80].

## Methods

### PEG hydrogels using UV photo-polymerization

PEG hydrogels were formed using free radical-based photo-polymerization, which is the most common method used to prepare biomimetic hydrogels [81]. To obtain 50 μl of 5wt%. PEG-Ac, 5 μl of 500mg/ml 4 arm-Ac-PEG (Mw = 10kDa, Laysan Bio, Inc., USA) were mixed with 2.5 μl of 200 mg/ml protease degradable peptide cross-linker GCRDVPMSMRGGDRCG (Mw = 2kDa, Genscript, USA), 1 μl of 5mg/ml photoinitiator (Irgacure 2959, Sigma Aldrich, USA) and 7.5 μl PBS. The thiolated crosslinker was added at a 2:1 molar ratio of acrylate:thiol. The PEG-Ac solution was cast into a PDMS mold of 50 μl hydrogel using UV irradiation at an intensity of 5 mW/cm^2^ (OmniCureR Series 1500, Excelitas Technologies Ltd, USA) for 5 min.

### Gel measurements with ferrule-top Chiaro Nanoindenter

Gel mechanics was evaluated using a nanoindentation device (Chiaro, Optics11, Netherlands) mounted on top of an inverted phase contrast microscope (Evos XL Core, Thermofisher, UK). All measurements were performed with the same cantilever with a stiffness *k* of 0.049 N/m and a spherical tip of 8 μm radius. For each sample, a 20 × 20 map with 10 μm spacing was recorded. Single indentations were acquired at the speed of 2 μm/s over a displacement of 10 μm. After every experiment, the probe was washed in ethanol 70% for 10 min.

### Cell culture

Human embryonic kidney (short: HEK-293T) cells were cultured at 37 $^\circ$C and 5% CO$_2$ in DMEM/F-12 culture medium supplemented with glutamine, 10% FBS and 1% penicillin-streptomycin and split using 0.05% trypsin-EDTA in PBS. For measurements, 300,000 cells per cm$^2$ were seeded in 2 cm$^2$ polydimethylsiloxane (short: PDMS, Specialty Manufacturing Inc, USA) wells in poly-D-lysine-coated culture dishes 2 days before the measurement to obtain a spatially confined fully confluent cell layer. The PDMS well was removed after one day, and prior to each measurement cells were incubated for 30 min at 37 $^\circ$C with physiological solution (140 mM NaCl, 5.4 mM KCl, 10 mM HEPES, 10 mM Glucose, 1 mM MgCl$_2$, 1.8mM CaCl$_2$, adjusted to pH 7.4 with NaOH) supplemented with 10 μM cytochalasin D, or 1 μM jasplakinolide and subsequently used for measurements at room temperature within 1 h without washing.

Cell culture reagents were obtained from Thermo Fisher Scientific, USA. Cytochalasin D and jasplakinolide were purchased from Abcam, UK. All other chemicals were purchased from Sigma-Aldrich, USA.

### Cell indentation measurements with FluidFM

Cell indentation measurements were performed with a FluidFM system consisting of a Nanosurf FlexAFM (Nanosurf AG, Switzerland) and a Cytosurge pressure controller (Cytosurge AG, Switzerland) on top of a Zeiss Observer inverted fluorescence microscope (Carl Zeiss AG, Germany). Cytosurge Micropipettes (Cytosurge AG, Switzerland) with 2 μm aperture and 0.3 N/m nominal spring constant were used as cantilevers with an integrated microfluidic channel. The spring constant was determined in air by thermal tuning and the Sader’s method [82] and the channel was filled with physiological solution containing 0.1 mg/ml of the blue fluorescent dye AMCA (7-amino-4-methylcoumarin, Sigma-Aldrich, USA) for blockage detection. Green fluorescent beads (Phosphorex Inc, USA) with 3 μm to 4 μm diameter were placed next to the confluent cell layer and attached to the cantilever tip by applying 800mbar suction pressure through the microchannel [74, 83]. Indentations were performed at 1 μm/s approach speed and up to 100nN force setpoint in grids of 5 × 5 points with 25 μm pitch.

### Data analysis

The obtained indentation data was analyzed with a custom python script based on the SciPy library [84] starting from the forward force-distance curves *F*(*z*) that were smoothed with a Savitzky-Golay filter [85]. The contact point was determined as the last peak of the ratio of variances as suggested in [43]. *F*(*z*) was transformed to a force-indentation curve $F(\delta )$ by subtraction of the contact point $z_0$ and division by the cantilever’s spring constant *k*. *F*(*z*) curves without a flat region or with high random variations were excluded. To obtain the standard Young’s modulus *E*, the average of the Hertzian fit of Eq. (1) to each curve was calculated. The conversion to the elasticity spectrum $E(\delta )$ of each single curve was performed by applying Eq. (5) with derivation by the Savitzky-Golay filter with a step size of 25 nm. For each data set, the elasticity spectra of all curves were averaged to obtain a mean elasticity spectrum $\left\langle E(\delta ) \right\rangle$. This curve was fit with the exponential decay in Eq. (8) to obtain values $E_0$, $E_b$ and $d_0$ for the cortex stiffness, bulk elasticity, and cortex thickness, respectively. The phenomenological factor $\Lambda$ of the exponential decay in Eq. (7) was determined to 1.74 by fitting the numerical $F(\delta )$ curve in Fig. 3b that was calculated by the FEA approach suggested in [52] and selecting the value to accommodate a physiologically relevant range (see Additional file 1: Fig. S2).

## Supplementary information

**Additional file 1.** Supplementary materials are provided including comments on the the impact of the rigid substrate on the ES, andthe variation of $\Lambda$.

## Data Availability

The datasets generated and/or analysed during the current study are available in the Enlighten repository of the University of Glasgow, DOI: 10.5525/gla.researchdata.1033
