# Supplementary material for: Elasticity spectra as a tool to investigate actin cortex mechanics
Source: J Nanobiotechnology. 2020 Oct 20;18:147. doi: 10.1186/s12951-020-00706-2 (PMC7576730; doi:10.1186/s12951-020-00706-2)
Supplement: Supplementary file 1 — Additional file 1. Supplementary materials are provided including comments on the the impact of the rigid substrate on the ES, andthe variation of $\Lambda$Λ. [file 12951_2020_706_MOESM1_ESM.docx]

Additional File 1

| **Elasticity Spectra as a Tool to Investigate Actin Cortex Mechanics** |
| --- |
| Ines Lüchtefeld^1^, Alice Bartolozzi^2^, Julián Mejía^3,4^, Oana Dobre^5^, Michele Basso^2^, Tomaso Zambelli^1^, Massimo Vassalli^5^  *^1^ Laboratory of Biosensors and Bioelectronics, ETH Zürich, Switzerland*  *^2^ Dipartimento di Ingegneria dell’Informazione, Università degli studi di Firenze, Firenze, Italy*  *^3^ Université Côte d'Azur, Nice, France*  *^4^ Dipartimento di Fisica, Università degli studi di Genova, 16100 Genova, Italy*  *^5^ James Watt School of Engineering, University of Glasgow, Glasgow, UK* |

**A Depth-dependence of ES**

When indenting into single cells or cell monolayers of a few µm thickness on top of a hard substrate, at a certain depth an increase of the elasticity spectrum can be observed as seen in Fig. S1b. This well-known phenomenon is associated to the presence of the stiff substrate, and it is typically suggested to keep the indentation depth below about 10% of the thickness of the sample. In fact, the thickness of the cell is normally not known, and having a direct method to visualize a reasonable fitting range is practical to direct the standard Hertz analysis as well. It is interesting to notice from Fig. S1b that the plateau for the apparent elastic modulus extends well beyond the suggested 10% (in this case, cells are thinner than 8-10µm). This is partially due to the balancing of two factors. The first is the already mentioned stiffer substrate, that tends to increase the modulus. The second, is the adoption of Eq. 4 to calculate the contact area. This equation assumes a parabolic shape for the indenter, and the area grows indefinitely, while in the real case the contact area is capped by the radius of the sphere. At some point, the error on this factor starts to play a role, which would reduce the apparent modulus, partially equilibrating the rise due to the substrate. In any case, ES appear a good tool to provide an estimate for the fitting range, that in the case of Fig. S1 can be apparently taken up to *δ* ≤ 2µm.


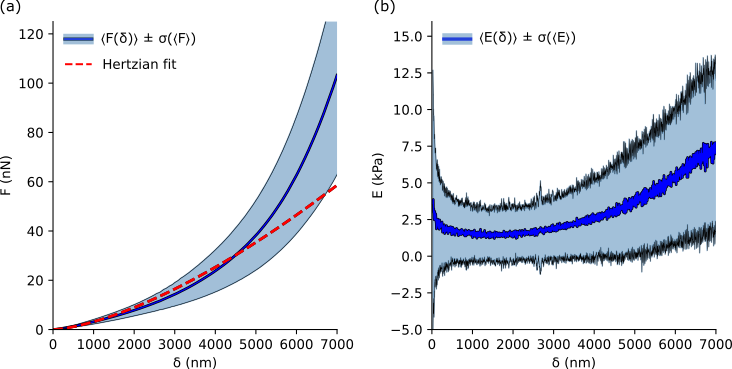


Figure S1 Indentation measurements for high indentations into a cell monolayer on top of a glass substrate. (a) Average of 315 force–indentation curves *F*(*δ*) (blue) and the Hertz fit (red) extended over the whole range. (b) Average elasticity spectrum of the same data set (blue).

**B Estimation of Λ**

The phenomenological factor Λ of Eq. 8 depends on the specific range of the physical parameters of the indented system. Using the FEA approach proposed in [50] we evaluated this coefficient for a range of physiologically expected values (see green boxes in Fig. S2):

5 ≤ E_0_ / E_b_ ≤ 10 and 200nm ≤ d_0_ ≤ 400nm . The dashed lines in Fig. S2 correspond to the choice $\bar{\boldsymbol{\Lambda}}=1.74$ for which the deviation of the apparent thickness from the real thickness is overall smaller than 10%.


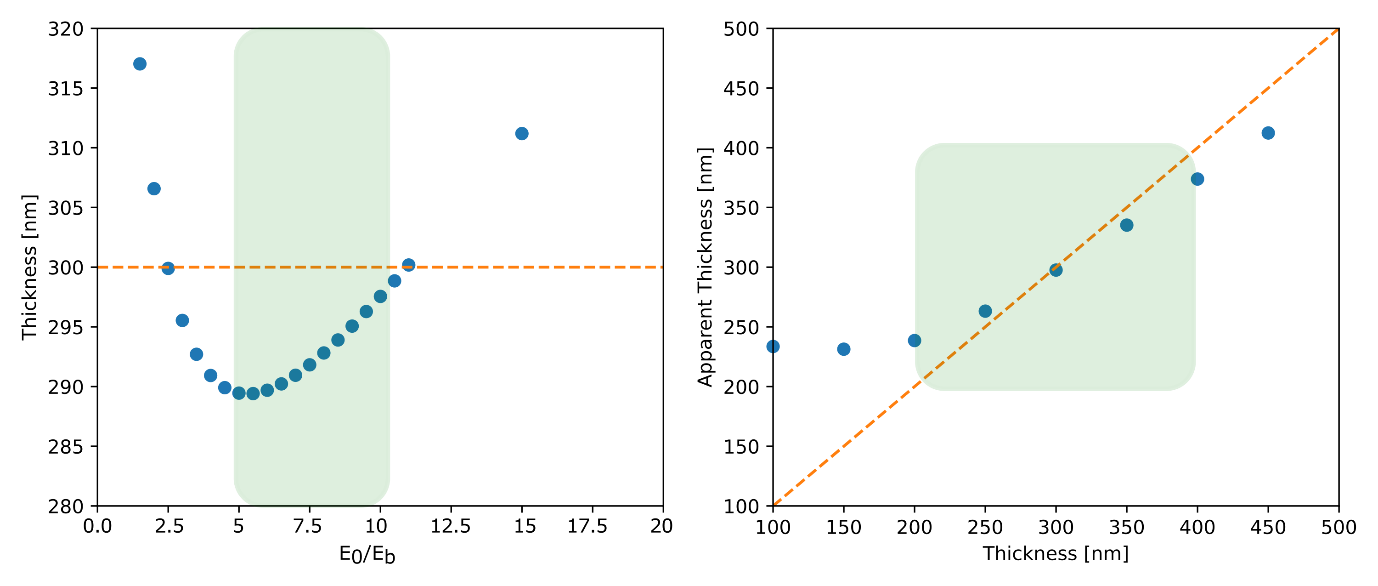


Figure S2 Numerical estimation of the apparent cortex thickness at variation of the input parameters to the FEA solution obtained from [50]. (a) Apparent cortex thickness as a function of the input cortex stiffness (blue), with the input cortex thickness (orange) and the physiologically expected range for *E*_0_ (green). (b) Apparent cortex thickness as a function of the input cortex thickness (blue), with the input cortex thickness (orange) and the physiologically expected range for *d*_0_.
